# Identification of New Genomospecies in the *Mycobacterium terrae* Complex

**DOI:** 10.1371/journal.pone.0120789

**Published:** 2015-04-01

**Authors:** Yun Fong Ngeow, Yan Ling Wong, Joon Liang Tan, Kar Wai Hong, Hien Fuh Ng, Bee Lee Ong, Kok Gan Chan

**Affiliations:** 1 Department of Medical Microbiology, Faculty of Medicine, University of Malaya, Kuala Lumpur, Malaysia; 2 Division of Genetics and Molecular Biology, Institute of Biological Sciences, Faculty of Science, University of Malaya, Kuala Lumpur, Malaysia; 3 Faculty of Veterinary Medicine, Universiti Putra Malaysia, Serdang, Selangor, Malaysia; National Institute of Infectious Diseases, JAPAN

## Abstract

Members of the *Mycobacterium terrae* complex are slow-growing, non-chromogenic acid-fast bacilli found in the natural environment and occasionally in clinical material. These genetically closely-related members are difficult to differentiate by conventional phenotypic and molecular tests. In this paper we describe the use of whole genome data for the identification of four strains genetically similar to *Mycobacterium* sp. JDM601, a newly identified member of the *M*. *terrae* complex. Phylogenetic information from the alignment of genome-wide orthologous genes and single nucleotide polymorphisms show consistent clustering of the four strains together with *M*. sp. JDM601 into a distinct clade separate from other rapid and slow growing mycobacterial species. More detailed inter-strain comparisons using average nucleotide identity, tetra-nucleotide frequencies and analysis of synteny indicate that our strains are closely related to but not of the same species as *M*. sp. JDM601. Besides the 16S rRNA signature described previously for the *M*. *terrae* complex, five more hypothetical proteins were found that are potentially useful for the rapid identification of mycobacterial species belonging to the *M*. *terrae* complex. This paper illustrates the versatile utilization of whole genome data for the delineation of new bacterial species and introduces four new genomospecies to add to current members in the *M*. *terrae* complex.

## Introduction

The *Mycobacterium terrae* complex (MTC) is made up of genetically closely-related slow-growing, nonchromogenic nontuberculous mycobacteria (NTM). This complex has expanded over the years to include *M*. *terrae* [1], *M*. *triviale* [2], *M*. *nonchromogenicum* [3], *M*. *hiberniae* [4], *M*. *kumamotonense* [5], *M*. *arupense* [6], *M*. *senuense* [7], *M*. *paraterrae* [8], *M*. sp. JDM601 [9], *M*. *engbaekii* [10], *M*. *longobardum* [10] and *M*. *heraklionense* [10]. Recently, however, *M*. *triviale* was excluded from the complex because of notable differences in the 16S rRNA sequence which included the presence of a short helix 18 that is generally considered a signature of rapidly growing mycobacteria [10].

Most MTC members are environmental bacteria of little clinical significance although they have been isolated occasionally from animal hosts [11] and human patients [12]. Nonetheless, in humans, chronic and debilitating infections have been reported [13] and multiple antibiotic resistance has contributed to the therapeutic problems posed by these infections [14].

Species differentiation for the MTC is traditionally based on DNA polymorphism in the 16S rRNA, *hsp*65 and *rpoB* genes and in the 16S-23S rRNA internal transcribed spacer [15,16]. With the greater accessibility of whole genome sequencing, however, these single and concatenated gene approaches are being surpassed by the use of whole genome data that can provide more comprehensive information for species and subspecies differentiation and the determining of phylogenetic relationships between bacterial strains. Numerous genome-wide nucleotide and protein-based approaches have been advocated, including the construction of phylogenetic trees based on the occurrence of protein-folds that collectively represent the 3-D structure of an organism [17]. As a result, many genonmospecies sharing a high degree of DNA relatedness have been recognized, leading to a greater appreciation of species complexity and enabling a more accurate definition of species boundaries. Despite technological advances, however, there are still taxonomic ambiguities and difficulties with the separation of strains into distinct taxa.

While conducting a study on the prevalence of tuberculosis in captive elephants [18], we recovered four NTM isolates from elephant trunk wash (UM_ Kg1, UM_Kg17, UM_Kg27, UM_NZ2, hereafter referred to as UM strains). As our routine diagnostic tests identified them variously as different species in the MTC, we analyzed the genomes of these isolates with a combination of different bioinformatics approaches, to study their phylogenetic relationship to *M*. sp. JDM601, the only member of the *M*. *terrae* complex with genome sequence data in the public domain (hereafter referred to as JDM601), as well as to other selected mycobacterial spp.

## Material and Methods

### Identification of mycobacterial isolates

Elephant trunk wash was collected and processed as described previously [18]. Acid-fast isolates on Middlebrook 7H10 agar were identified as NTM by a negative reaction for *M*. *tuberculosis* MPB64 antigen in the Tibilia test (*TibiliaTM* TB, Genesis, China). For NTM species identification, DNA was extracted by boiling a mycobacterial suspension at 100°C for 30 min. followed by centrifuging at 16,100g for 2 min. The supernatant was used for 16S rRNA-, *rpoB-* and *hsp65-*based PCRs as described in Lane [19], Adekambi [20] and Telenti [21], respectively (S1 Table). PCR products were purified using the QIAquick PCR Purification kit (QIAGEN, Germany) and outsourced for Sanger sequencing. The resulting DNA sequences were searched against the NCBI non-redundant (nr) nucleotide database using BLAST web server [22]; *hsp65* sequences were further submitted to a Web-accessible database (http://msis.mycobacteria.info) [23]. The most probable species for each isolate was identified based on the nucleotide sequence similarity with reference strains.

### Whole genome sequencing

A Nextera DNA sequencing library was prepared according to the Illumina Nextera protocol. In short, 40 ng of genomic DNA was tagged and fragmented using Nextera DNA Sample Prep Kit (Illumina Inc., CA), followed by DNA clean-up using DNA Clean & Concentrator (ZymoResearch, CA). Next, adaptors were added using Nextera Index Kit (Illumina Inc., CA) followed by PCR clean-up using Agencourt AMPure XP (Beckman Coulter, CA). Finally, sequencing was performed using the Illumina HiSeq 2500 system, on rapid run mode.

Raw sequences were subjected to the trimming of adapters and low quality reads based on Q25, using Trimmomatics [24] and Sycthe (https://github.com/vsbuffalo/scythe). SGA [25] was used for error correction and the sequencing quality of the reads was investigated using FastQC [26]. The data were then assembled using CLC Genomics Workbench v.7.0, with kmer size of 45. Only contigs larger than 200bp were used for downstream analysis. Gene prediction was accomplished with Prokaryotic Dynamic Programming Genefinding Algorithm (PRODIGAL) Version 2.60 [27] and the annotation of predicted CDSs was by homology search against the nr database. tRNA and rRNA were predicted using Aragon [28] and RNAmmer [29] respectively.

### Phylogenetic and phylogenomic analyses

We constructed Maximum Likelihood (ML) phylogenetic trees based on the 16S rRNA, *rpoB* and *hsp65* genes to study the relationship between the UM strains and other members of the MTC comprising JDM601, *M*. *terrae*, *M*. *kumanotonense*, *M*. *senuense*, *M*. *nonchromogenicum*, *M*. *longobardum*, *M*. *heraklionense*, *M*. *arupense*, *M*. *hiberniae* and *M*. *engbaekii*. The marker gene sequences of UM strains were obtained from their respective genomes, while those of the comparison strains were downloaded from the NCBI taxonomy database. The single gene and supermatrix trees were constructed with 1000 bootstrap replications, using MEGA software [30] version 5.2 with the Hasegawa-Kishino-Yano model for the 16S rRNA tree, the Tamura 3-parameter model for the *hsp65* tree, and the Tamura-Nei model for the *rpoB* and the three gene concatenated trees. *Nocardia farcinica* (NC_006361) was included as the out-group.

Subsequently, we compared UM strains against mycobacterial spp. with whole genome sequences deposited in the NCBI Genome database up to 9^th^ April 2014. These genomes represent the *M*. *terrae* complex (JDM601), rapid-growing mycobacteria (RGM) (*M*. *smegmatis*, *M*. *abscessus* subsp. *bolletii*, *M*. *chelonae*, *M*. *fortuitum* subsp. *fortuitum*, *M*. *septicum*,) and slow-growing mycobacteria (SGM) (*M*. *marinum*, *M*. *ulcerans*, *M*. *avium* subsp. *paratuberculosis*, *M*. *parascrofulaceum*, *M*. *liflandii*, *M*. *avium*, *M*. *intracellulare*) (S2 Table). Four ML trees were constructed based on the full length 16S rRNA, *rpoB* and *hsp65* genes separately and concatenated. All trees were constructed with 1000 bootstrap replications and visualized with MEGA 5.2 software, using the Tamura-Nei model for the 16S rRNA tree and the General Time Reversible model for the *rpoB*, *hsp65* and concatenated trees.

We then made two more comparisons, one using genome-wide orthologous genes and another using a genome alignment-free approach, to find single nucleotide polymorphisms (SNP). For the first approach, the 1084 single copy orthologous CDSs generated from OrthoMCL [31] with expected value of 1e-5 and inflation value of 1.5, were aligned with MUSCLE [32]. After using trimAL [33] to select informative regions, ProtTest [34] was used to select the best fit ML model with the Akaike Information Criterion framework (AIC) [35]. The resulting model (LG+I+G+F) was implemented in PhyML 3.0 [36]] to generate an ML tree with 100 bootstrap replications.

In the SNP-based comparison, our UM genomes together with completed and draft sequences downloaded from GenBank were subjected to a series of preprocessing processes to generate a FASTA input file that is compatible with the analysis software. The kchooser script was used to select an optimum value of k-mer size for the data set. Jellyfish in the kSNP package was used to enumerate a list of k-mers at optimum k-value for each genome in the data set. The sequence variation of the data set was evaluated and the fraction of core-k-mers at optimum k-value was identified. kSNP was used to search for putative SNP loci in the data set and the identified SNP alleles were concatenated into a string. Multiple sequence alignment was produced for each nucleotide in the SNP matrix with MUSCLE. The best fit nucleotides substitution model was tested in MEGA version 5.2 and the Kimura-2 model was selected to generate the ML tree with 100 bootstrap replications.

### Inter-strain comparisons with ANI, TETRA, shared gene families and syntenic analysis

To further examine the degree of genetic relatedness between the UM strains and JDM601, we conducted analyses with average nucleotide identity (ANI) [37] and tetra-nucleotide usage patterns, using TETRA (http://www.megx.net/tetra) [38]. To obtain the ANI, all conserved genes between a pair of genomes were determined by whole-genome sequence comparisons using the BLAST algorithm. Genes from the query genome were considered conserved when they had a BLAST match of at least 30% overall sequence identity and the aligned region being at least 70% of its length in the reference genome. Strains sharing more than 94% ANI are considered belonging to the same species. In the TETRA analysis, the correlation indices of the tetra-nucleotide signatures between pair-wise genomic comparisons were calculated. Correlation coefficient values above 0.99 suggest the probability of two strains being from the same species.

In addition, a Venn diagram was drawn to show the degree of gene sharing among the UM strains and JDM601. Homologous genes were identified as genes sharing >50% protein sequence identity. For the analysis of synteny, we used Progressive Mauve software [39] for five-way genome alignments to identify Locally Collinear Blocks (LCBs) representing large scale differences generally due to evolutionary events such as gene loss, rearrangements, duplication, and horizontal transfer. The contigs from UM strains were reordered using JDM601 as the reference. Default parameters were used and alignments were visualized in LCB color scheme.

### Signature genes

To identify candidate signatures for the MTC, we looked for conserved genes in selected non-terrae *Mycobacterium* spp. and contrasted these genes against those that are conserved only in JDM601 and UM strains. With an initial 50% cut-off, 6677 gene clusters were found. After the exclusion of genes not in the *M*. *terrae* group (JDM601, UM strains), 160 gene clusters remained. After further bidirectional BLAST searches in the NCBI nr and biocyc (http://biocyc.org/) databases, only five putative protein coding genes remained that are unique to the UM strains and JDM601.

## Results

### Identification of isolates

The four UM strains were cultured on 7H10 Middlebrook agar. After 8–14 days of incubation at 36°C, smooth to rough, white to pale buff colonies appeared that stained positive for acid-fast bacilli but were negative in the Tibilia test for *M*. *tuberculosis*. PCR-sequencing of the 16S rRNA, *hsp65* and *rpoB* genes identified the strains as different members of the MTC (*M*. *arupense*, *M*. *engbaekii*, *M*. *terrae*, *M*. *paraterrae*, *M*. *hiberniae* and *M*.sp. F1–10193) with 96–100% sequence similarity (Table 1).

**Table 1:** Identification of UM strains using marker gene sequences.

Strain	Growth and colonial morphology on Middlebrook 7H10 agar	*hsp65* PCR (440bp)	16S rRNA PCR (724bp)	*rpoB* PCR (918bp)
UM_Kg1	Slow; 1mm; non-pigmented; smooth to dry	*M*. *hiberniae* (97)	*M engbaekii* (100)	*M engbaekii* (98)
UM_NZ2	Intermediate; 5mm; non- pigmented; dry; irregular edge	*M*. *arupense* (96)	*M*. *arupense* (99)	*M*. sp. F1–10193* (99)
UM_Kg17	Intermediate; 1mm; smooth-dry; turned pale pink at 3 weeks of incubation	*M*. *arupense* (98)	*M arupense* (99)	ND
UM_Kg27	Intermediate; non-pigmented; 1mm; smooth-dry	*M*. *arupense* (96)	*M paraterrae* (99)	*M terrae* (99)

*A new sequevar of the *M*. *terrae* complex described by Tortoli et al. [10]

ND, not done

### Genome overviews

The number of Illumina sequencing reads generated for the UM genomes ranges from 5,107,720 to 24,102,740, resulting in 192 to 442 contigs with an N50 contig size of 25,337 to 67,651 bp. The genome sizes are from 4.2Mb (UM_Kg17) to 5.1Mb (UM_NZ2), with 67.4–68.5% G+C content. We predicted 3923 to 4811 putative coding sequences and 54 to 64 RNAs (S3 Table).

**Fig 1:**
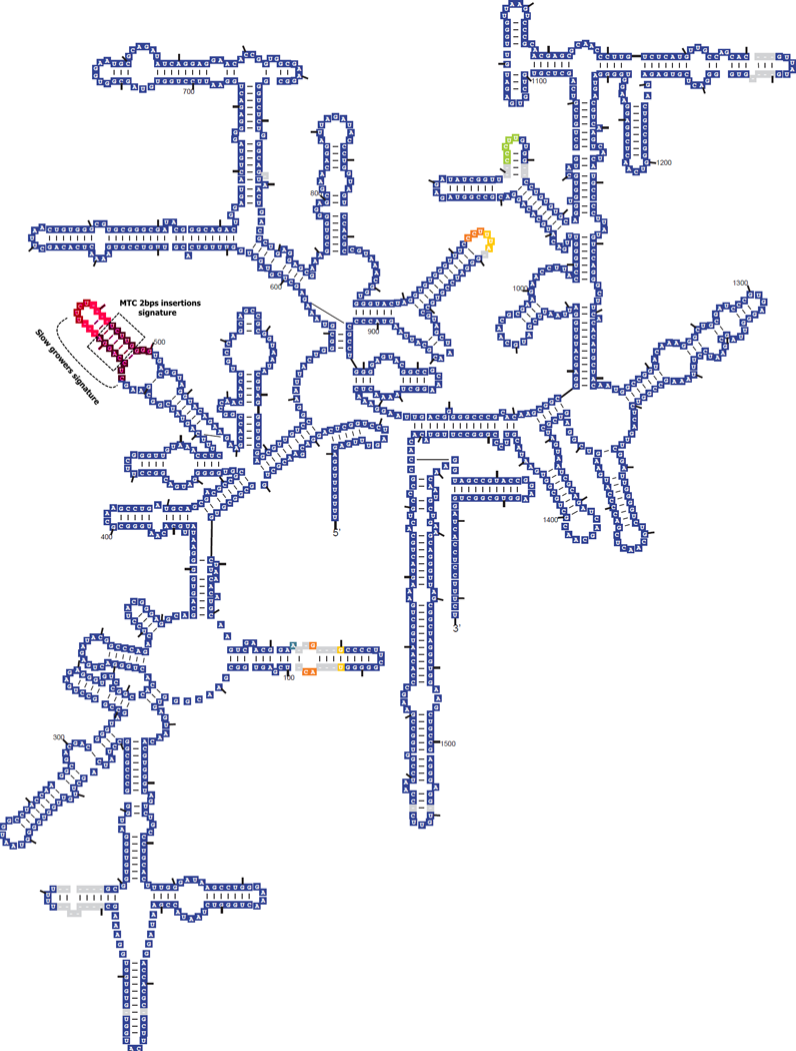
Secondary structure of 16S rRNA gene showing an extended helix 18 (455^th^ to 477^th^ in the boxed area), in UM strains.

All four UM strains show two characteristic features that distinguish the SGM from the RGM. Firstly, like other SGM, there appears to be only one copy of the rRNA operon in each UM strain; this is in contrast to *M*. *terrae* and JDM601 which were both reported to have two copies of the rRNA operon, despite being slow growers [9]. Secondly, all UM strains show the signature of SGM which is a long version of helix 18 in the 16S rRNA gene [40]. In addition, UM strains have the 2bp insertion in helix 18 (Fig. 1) that has been reported to be unique in the MTC [41].

Using the Phage Search Tool (PHAST) web server (http://phast.wishartlab.com) [42], we identified putative prophages in all UM strains. The largest genome, UM_NZ2, carries five prophages which, together, make up 187.6kb (3.7%) of the genome (S4 Table). Biological functions such as secretion, transcription regulation and stress response can be assigned to the genes in all the prophages. However, we failed to identify any match with phages in the PHAST phage library, the NCBI nucleotide database and the Mycobacteriophage Database at http://phagesdb.org/blast/. Thus, the prophages in our UM strains may be novel, a possibility that has to be verified with phage induction and characterization.

### Phylogenetic and phylogenomic analyses

**Fig 2:**
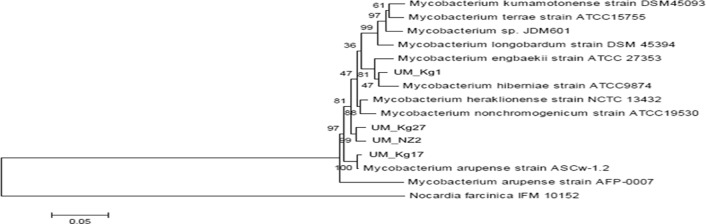
Supermatrix phylogenetic tree for members of the *M*. *terrae* complex, based on concatenated 16S rRNA, *rpoB* and *hsp65* genes (1931bp).

The individual and concatenated phylogenetic trees based on partial sequences of the three marker genes (*hsp65*, *rpoB* and 16S rRNA) consistently show the UM strains among members of the MTC (S1–S3 Figs.; Fig. 2; S5–S7 Tables). In the similarity matrix constructed for the 16S rRNA (S5 Table), UM_Kg1 is 100% similar to *M*. *engbaekii* ATCC27353, UM_Kg17 is 100% similar to 9 of 10 strains of *M*. *arupense* while UM_Kg27 and UM_NZ2 are 99.77% and 99.65% similar to *M*. *paraterrae* 052522, respectively. In the concatenated tree (Fig. 2), although the nearest neighbours to UM strains have changed, they are still members of the MTC complex.

**Fig 3:**
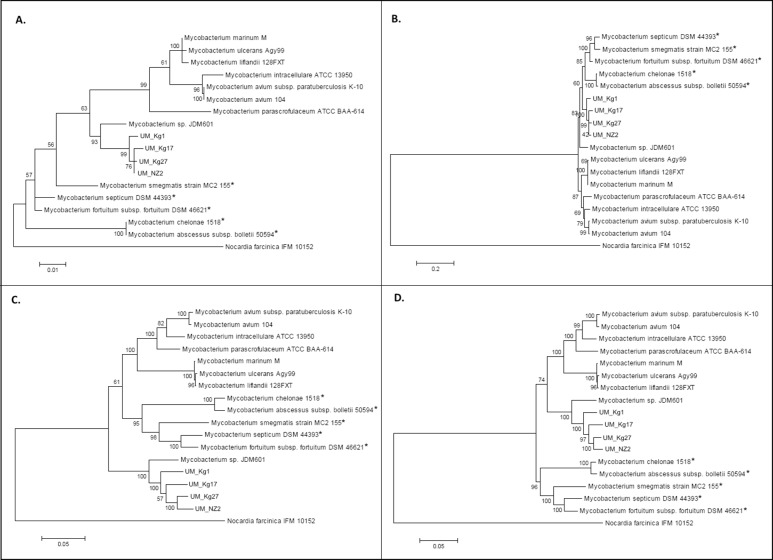
Phylogenetic trees to show the relationship of UM strains with SGM and RGM spp. **based on** A) the 16S rRNA gene; B) the *hsp65* gene; C) the *rpoB* gene; and D) concatenation of 16S rRNA, *hsp65* and *rpoB* genes. RGMs are indicated by an *.

When compared to other RGM and SGM, using entire marker gene sequences extracted from whole genome data, the four UM strains are invariably clustered with JDM601 in a distinct clade among the SGM species (Fig. 3). Two strains, UM_NZ2 and UM_Kg27, are the closest neighbors in seven of the eight trees constructed, suggesting a high possibility that they may belong to the same species.

**Fig 4:**
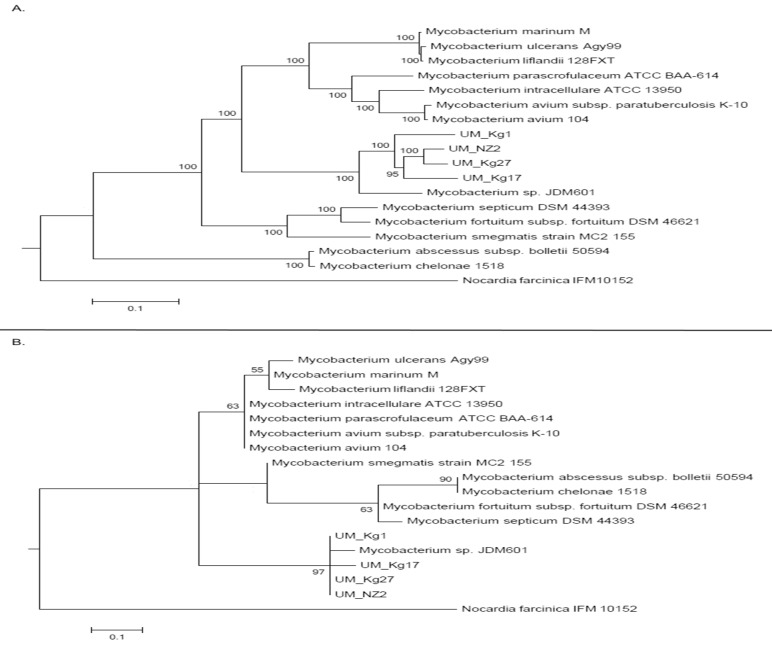
Phylogenetic trees based on whole genome sequences. A) constructed with genomic orthologous genes; B) constructed with genome-wide SNPs.

The clustering of UM strains and JDM601 is again obvious in the trees constructed with orthologs and SNPs. Both trees show almost identical topology, indicating a high concordance in the amino acid and nucleotide sequences of the species under study (Fig. 4).

### Further inter-strain comparisons

While the phylogenetic and phylogenomic analyses suggest that the UM strains are different genomospecies in the MTC, further inter-strain comparisons are necessary to refine their taxonomic relationships. For this purpose, we used the ANI together with TETRA. Unfortunately, although the tetranucleotide frequency correlation coefficients (Table 2) indicate a strong likelihood that the UM strains and JDM601 all belong to the same species, the ANI values are all below the 94% threshold for strains to be classified as belonging to the same species (Table 3). As in the phylogenetic analysis, UM_NZ2 and UM_Kg27 appear to be the most closely-related genetically, sharing the highest ANI (91.76%) and TETRA (0.99799) values.

**Table 2:** Results of TETRA analysis for UM strains and JDM601.

	Tetranucleotide frequency correlation coefficient values				
	JDM601	UM_Kg17	UM_Kg1	UM_Kg27	UM_NZ2
JDM601	---	0.9912	0.99174	0.99404	0.99259
UM_Kg17	0.9912	---	0.9908	0.99371	0.99273
UM_Kg1	0.99174	0.9908	---	0.9946	0.99164
UM_Kg27	0.99404	0.99371	0.9946	---	0.99799
UM_NZ2	0.99259	0.99273	0.99164	0.99799	---

**Table 3:** Results of ANI analysis for UM strains and JDM601.

	ANI values in percentage (%)				
	JDM601	UM_Kg17	UM_Kg1	UM_Kg27	UM_NZ2
JDM601	---	84.77	85.2	85.49	85.53
UM_Kg17	84.93	---	85.89	86.81	86.64
UM_Kg1	85.2	85.47	---	86.65	86.76
UM_Kg27	85.5	86.54	86.65	---	91.75
UM_NZ2	85.54	86.39	86.76	91.76	---

**Fig 5:**
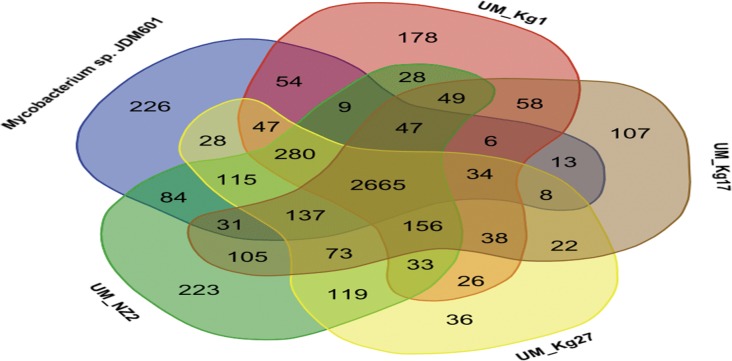
Venn diagram for the genomes of UM strains and JDM601, showing shared and strain-specific genes.

The Venn diagram constructed for the four UM strains and JDM601 show them sharing a large number of gene families (Fig. 5). More than half (2665 of 5035 gene families) are identified as the core genome, 2144–2334 (42.6–46.4%) are shared with at least one other strain and only 36–226 (0.7–6.7%) appear to be strain-specific. The UM strains share 58.4% (UM_Kg17) to 66.9% (UM_NZ2) of their genes with JDM601, and 60.6% to 71.1% among themselves (Fig. 5). The two genomes sharing the highest ANI and TETRA values (UM_NZ2 and UM_Kg27) show the greatest (71.1%) identity in gene families. Likewise, JDM and UM_Kg17 share the lowest ANI (84.77%) as well as the lowest gene family identity (58.4%).

**Fig 6:**
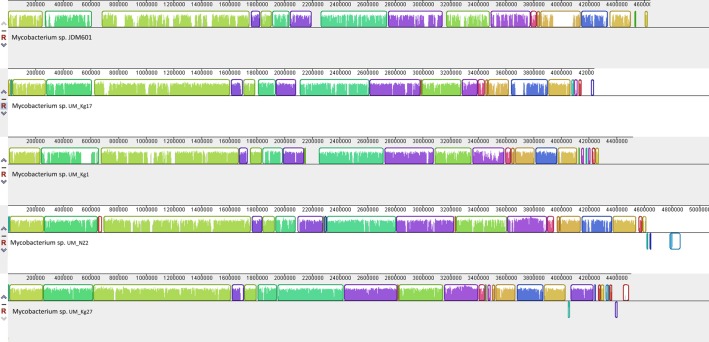
Multiple sequence alignment of UM strains and JDM601, using Progressive MAUVE and default parameters. Coloured blocks are collinear and homologous regions. Non-coloured areas are unaligned sequences that may be genome-specific. Inverted regions are shown as blocks below a genome’s centre line.

Syntenic analysis for the UM strains and JDM601 show putative homologous matches over much of the genome sequences compared (48% to 62% of the JDM601 genome appear conserved in the UM strains), suggesting a high degree of gene-order conservation (Fig. 6). Nevertheless, a number of prominent variations are seen that suggest horizontal gene transfers and other events of evolutionary change. Although the four UM strains are more syntenic to one another than to JDM601, variations are still seen among them, such as translocations and inverted sequences in UM_Kg27 and UM_NZ2. The prophage regions we identified appear as genome-specific sequences, indicating that horizontal transfers occurred after the separation of UM strains from the progenitor of the MTC.

### Candidate *M*. *terrae* signatures

Phylogenetically-related genomes have been observed to share similar genomic signatures. Among the *M*. *terrae* complex, a 2bp addition to the long helix 18 in the 16S rRNA has been reported to be specific to members of this complex [41]. We used other conserved genomic regions in our search for additional signatures and found five predicted protein coding sequences in UM strains and JDM601 that are not observed in selected non-terrae mycobacterial spp. (Table 4). These hypothetical proteins are potentially useful as diagnostic markers for the determination of species in the *M*. *terrae* complex and for the rapid detection of these species in a mixed bacterial community.

**Table 4:** Amino acid and corresponding nucleotide sequences for candidate ***M*. *terrae*** signatures.

Gene	Sequence
JDM601_0345	MAGVIAALACTLGCCLPAILVALGVGASGAAGMGHAAHGAGESRGALLDLLHRVSPALLIASIALVAGAFAMRRRAAVLPALLAGVVLYLSVHGQTDPAVMYAGMAIGYGVWIGLYFWTRRSAQACEHEAGGVGAEVGAAHREPPIDPVQGQ
	GTGGCGGGCGTTATCGCCGCACTGGCCTGCACGCTGGGGTGCTGCCTGCCGGCGATCCTGGTGGCACTCGGGGTCGGCGCATCCGGGGCCGCAGGCATGGGCCATGCGGCGCACGGCGCCGGTGAGTCTCGGGGCGCCCTGCTCGACCTGTTGCACCGGGTCAGCCCGGCGCTGCTGATCGCGTCGATCGCGCTGGTCGCCGGCGCATTCGCTCGACCTGTTGCACCGGGTCAGCCCGGCGCTGCTGATCGCGTCGATCGCGCTGGTCGCCGGCGCATTCGCGATGAGGCGACGCGCGGCGGTGCTGCCCGCGCTGTTGGCCGGGGTCGTGTTGTACCTCAGCGTTCACGGTCAGACCGATCCCGCCGTGATGTATGCCGGTATGGCCATCGGCTACGGCGTCTGGATCGGCCTCTATTTCTGGACGCGCCGGTCAGCCCAGGCCTGCGAGCACGAGGCGGGTGGCGTAGGCGCCGAGGTCGGCGCGGCTCACCGGGAGCCGCCCATCGACCCGGTCCAGGGTCAGTGA
JDM601_0373	MLALSCCLLAAPAAHADFDDLLDTLLGAGVDVDPGALPADSADIGDLGVLQDPLSQLDQLFHDSPSSADGTPEEPAEPAPEAPTDDPSDSTAGGEHSSESGGSNNLPSLPKFGMPGSGSGGSGGGPGGGSGGGPGGSSPGAAKTKANTSGSNGTGGAPVTPAPQR
	GTGTTGGCGTTGAGTTGCTGCCTGCTGGCCGCGCCGGCCGCGCATGCCGACTTCGACGACCTGCTCGACACCCTGCTCGGCGCCGGTGTCGATGTGGACCCGGGCGCTCTGCCGGCAGATTCGGCCGATATCGGCGACCTCGGTGTCCTGCAGGACCCGCTGAGCCAGCTCGACCAGTTGTTCCACGACTCGCCGAGCTCCGCCGACGGCACTCCCGAGGAGCCCGCCGAGCCGGCCCCCGAAGCGCCGACCGACGACCCGTCCGACTCGACCGCGGGTGGCGAACACAGCAGCGAGAGCGGCGGTTCCAACAACCTGCCGAGCCTGCCGAAGTTCGGCATGCCCGGCAGCGGCTCCGGCGGCAGCGGTGGCGGCCCCGGCGGCGGCAGCGGCGGCGGACCCGGCGGTTCCAGCCCGGGCGCCGCGAAGACCAAGGCCAACACCAGCGGCAGCAACGGCACCGGCGGCGCGCCGGTGACGCCGGCCCCACAGCGGTAA
JDM601_1987	MSRIRKYLTVAAGAAAGLFLGALASSSAATADTAPINPGLPGVVEQMVASSTAIPQQLLQTTTSALSGTPLAPAASPAQSPIATATLNVPQTTTPASQPTGLPGLSGLPANLSSVLPFPMPNFGGTTPVAAAPTTMVPGAFAPTAPVTPMEVMLIPGLP
	ATGTCACGAATCCGTAAGTACCTCACCGTTGCGGCCGGCGCCGCCGCGGGACTGTTCCTGGGGGCACTCGCGTCCAGCAGCGCCGCCACCGCGGACACCGCTCCGATCAACCCGGGGCTGCCCGGTGTGGTCGAGCAGATGGTCGCGTCCTCGACGGCCATTCCGCAGCAGTTACTGCAGACCACCACCTCCGCCCTGAGTGGAACGCCGCTGGCGCCCGCCGCGTCGCCGGCGCAGTCACCGATCGCCACCGCCACACTCAACGTGCCGCAGACCACGACGCCGGCCAGCCAGCCCACCGGCCTGCCCGGGCTGTCCGGTCTCCCGGCCAACCTGAGTTCGGTGCTGCCGTTCCCGATGCCGAACTTCGGCGGCACGACACCGGTGGCGGCCGCACCGACCACGATGGTCCCCGGCGCCTTCGCGCCGACGGCTCCGGTGACGCCGATGGAGGTCATGCTGATTCCCGGCTTGCCCTGA
JDM601_2719	MNATLRPFALAGAAIIGATAIAATPVVVVPVSVPTPVIELTADAGGAFEGALGSLGDSLGNLLSSLDLSEILGDLFSNLNLSEIFGDFFANLNLSEMFGDFFANFDLSGIFTGITDFFANFDLSGIFTGITDFFANFDLSGIFTGITDFFADFDLSGIFTGITDFFADFDLSGIFTGLGDFFADFDLSELLGGLDLSGLLDDLFGGL
	ATGAACGCCACCCTTCGTCCGTTTGCCCTGGCGGGCGCCGCGATCATCGGCGCCACCGCAATCGCCGCCACACCCGTGGTGGTCGTACCCGTGAGCGTGCCGACACCGGTCATCGAGCTCACTGCCGACGCCGGCGGCGCGTTCGAGGGTGCGCTCGGCTCGCTCGGCGACAGTTTGGGCAACCTGCTGAGCAGCCTGGATCTGTCGGAGATCCTGGGCGACCTCTTCAGCAACCTGAATCTGTCGGAGATCTTCGGCGACTTCTTCGCCAACCTGAATCTGTCCGAAATGTTCGGCGACTTCTTCGCCAACTTCGACCTGTCGGGCATCTTCACCGGCATCACCGACTTCTTCGCCAACTTCGACCTGTCGGGCATCTTCACCGGCATCACCGACTTCTTCGCCAACTTCGACCTGTCGGGCATCTTCACCGGCATCACCGACTTCTTCGCCGACTTCGACCTGTCGGGCATCTTCACCGGCATCACCGACTTCTTCGCCGACTTCGACCTGTCGGGCATCTTCACCGGCCTCGGCGACTTCTTCGCCGACTTCGACCTGTCGGAGCTCCTGGGCGGCCTGGATCTGTCCGGCTTGCTCGACGACCTGTTCGGCGGTCTGTAA
JDM601_3872	MAYADEPTLAAEQQAAEQQAAAEQQVAEQHAEQATTAGATTAGAGGMGASIGSSMAMMGPMMAMYAPMMLAPMLMSGVTSLVGGAPATGAEAASAAATDFAGSAAATDAWGLAAPIASDLANLDLTLDPDVLTGALTDTGAMLGADVADATADAAGQAAADLAGQTAADTAGDLAASLGGELPGIAAETGVNVGGEIACGIIGGIFGVC
	TTGGCGTACGCCGATGAGCCGACGCTGGCCGCTGAGCAGCAGGCGGCTGAGCAGCAGGCAGCAGCCGAGCAGCAGGTAGCCGAGCAGCACGCCGAGCAGGCCACCACGGCGGGTGCCACGACCGCCGGCGCCGGCGGCATGGGCGCCTCCATCGGCAGCTCGATGGCGATGATGGGCCCGATGATGGCGATGTACGCGCCCATGATGCTCGCACCGATGCTGATGTCGGGTGTGACCAGTCTTGTCGGGGGAGCCCCGGCCACCGGCGCGGAGGCCGCTTCGGCCGCGGCTACGGATTTCGCCGGTTCGGCGGCGGCCACCGACGCCTGGGGCCTGGCGGCTCCGATCGCCAGTGACCTGGCCAATCTCGATCTGACCCTCGACCCTGACGTGTTGACGGGTGCGCTCACCGACACCGGTGCCATGCTGGGCGCCGACGTTGCCGACGCTACGGCCGATGCCGCCGGCCAGGCGGCCGCGGATCTGGCCGGCCAGACCGCGGCCGACACCGCGGGCGACCTTGCCGCCAGCCTGGGCGGCGAGCTGCCGGGAATCGCCGCCGAAACCGGCGTGAACGTCGGTGGCGAGATCGCCTGCGGCATCATCGGCGGCATCTTCGGCGTCTGCTGA

## Discussion

Whole genome shotgun sequencing (WGS) is making inroads into clinical microbiology with the promise of unlimited genetic information for the identification and characterization of pathogens as well as the prediction of resistance to antimicrobials. The success of WGS-based diagnostics will depend very much on the availability, size and quality of genomic databases. Unfortunately, for many bacterial taxa, there are still too few genomes to provide a comprehensive database for accurate pathogen recognition and analysis.

In this study, we used different approaches to determine the taxonomic status of four strains of RGM which we were unable to resolve into definite species with conventional tests. The results indicated a high degree of consistency but also some discrepancies with the use of different analytical methods. With our routine diagnostic partial sequencing of the 16S rRNA, *hsp65* and *rpoB* genes, each of the four strains had more than one species identification, albeit, all from the MTC. Their species designation is largely supported by our concatenated gene-based phylogenetic analysis for representative members of the MTC. With WGS, we were able to extract full length marker gene sequences as well as genome-wide orthologous genes and SNPs for the comparison of phylogeny. Our results showed a consistent clustering of UM strains with JDM601, and, at 100% coverage (1523bp), all four UM strains shared about 98% 16S rRNA sequence identity with JDM601. These results are suggestive of the UM strains being subspecies of JDM601. This possibility is also indicated by the TETRA correlation coefficients which are all over 0.99. However, the ANI values which are all below 94%, indicate that there ought to be five different species assignments for the five strains.

The ANI has been recognized to be one of the most reliable measurements of genomic relatedness between bacterial strains. The ANI threshold range (95–96%) for species demarcation previously set by comparison with DNA-DNA hybridization (DDH) values, was confirmed by Kim et al. [43] who investigated the distribution of ANI values in over six thousand prokaryotic genomes. These authors also showed, with over a million comparisons, that this ANI threshold corresponds to 98.65% 16S rRNA sequence similarity. Nonetheless, exceptions to the rule do occur. It has been reported that even strains showing close to 100% 16S rRNA identity can be assigned to different species [44] and conversely, at the 94% ANI cutoff, many species could share less than 90% of their genes [45].

Oligonucleotide frequencies within DNA sequences are known to exhibit species-specific patterns [46]. TETRA analyzes tetranucleotide usage patterns in genomic fragments. This method of analysis has been reported to be more reliable than the G+C content as a measure of sequence relatedness and is useful not only for the classification of bacteria, but also for the estimation of phylogenetic relationships among closely related species [47]. Furthermore, since oligonucleotide frequency can be determined without the need for multiple alignments of homologous sequences, it can be applied on genomes that have not been previously aligned and annotated.

A comparison of four members of the *M*. *tuberculosis* complex (*M*. *tuberculosis*, *M*. *africanum*, *M*. *canettii* and *M*. *bovis*) showed the expected agreement of ANI (>97%) and TETRA (0.99965–0.99994) values (data not shown). A possible explanation for the discrepancy between the results of our ANI and TETRA analyses for UM strains is the hypothesis that closely-related bacteria, subjected to the same environmental pressures in a complex habitat, can evolve to different species without losing their genetic signatures. Foerstner et al. [48] observed distinct and narrow G+C distributions among closely-related bacterial sequences present in the same environment, in contrast to a wider range of G+C content among closely-related sequences from different environments. They attributed this difference in G+C content to the influence of external environmental factors on the nucleotide compositions of co-evolving bacteria in a bacterial community. Konstantinidis and Tiedje [45] similarly postulated that differences in the ecology of bacterial strains can lead to differences in the conserved gene content among strains in the same species. Although our UM strains are all isolated from elephant trunk wash, the elephant hosts are from different countries of origin and are maintained in different conservation centres. It is possible that the UM strains, in their respective ecological niches, underwent genome size expansion and genome diversification by acquiring foreign sequences like prophages, but still retained their MTC-specific tetranucleotide signature.

Bacterial identification and pathogen detection are essential for the correct diagnosis and treatment of infections. Molecular diagnostic laboratories have long depended on 16S rRNA sequences for pathogen identification. Our results reaffirm the inadequacy of the single gene approach and illustrate the advantages of using genome-based methods that allow multifaceted characterization of strains and exhaustive inter-strain comparisons for more reliable species and subspecies definition. Comparative genomic analysis of the MTC is currently limited by the scarcity of published genomic sequences. With the introduction of four new genomospecies and five potential genetic signatures, we are enlarging the MTC database for future analyses. Before whole genome sequencing becomes affordable for the routine diagnostic service, PCR assays based on marker genes, such as the MTC signatures identified in this study, can be implemented easily for the rapid identification of bacterial pathogens in clinical specimens.

## Nucleotide Sequence Accession Numbers

The GenBank database accession numbers for UM strains are:

Mycobacterium sp. UM_Kg1 JRMK00000000

Mycobacterium sp. UM_Kg17 JRML00000000

Mycobacterium sp. UM_Kg27 JRMM00000000

Mycobacterium sp. UM_NZ2 JRMN00000000

## Supporting Information

S1 Fig The 16S rRNA gene (852bp) tree for UM strains and other species in the *M*. *terrae* complex. (TIF)

S2 Fig The *hsp65* gene (399bp) tree for UM strains and other species in the *M*. *terrae* complex. (TIF)

S3 Fig The *rpoB* gene (680bp) tree constructed for UM strains and other species in the *M*. *terrae* complex. (TIF)

S1 Table Details of single gene amplifications by PCR. (DOCX)

S2 Table Accession numbers for genome sequences used in the phylogenetic analyses based on SNPs and orthologous genes. (DOCX)

S3 Table Illumina Hiseq 2500 assembly details for four UM strains of mycobacteria. (DOCX)

S4 Table Characteristics of prophages in UM strains. (DOCX)

S5 Table 16S rRNA similarity matrix (%) between UM strains and reference strains. (DOCX)

S6 Table *hsp65* similarity matrix (%) between UM strains and reference strains. (DOCX)

S7 Table *rpoB* similarity matrix (%) between UM strains and reference strains. (DOCX)
