# Supplementary figures and images for: Identification of New Genomospecies in the Mycobacterium terrae Complex
Source: PLoS One. 2015 Apr 1;10(4):e0120789. doi: 10.1371/journal.pone.0120789 (PMC4382200; doi:10.1371/journal.pone.0120789)

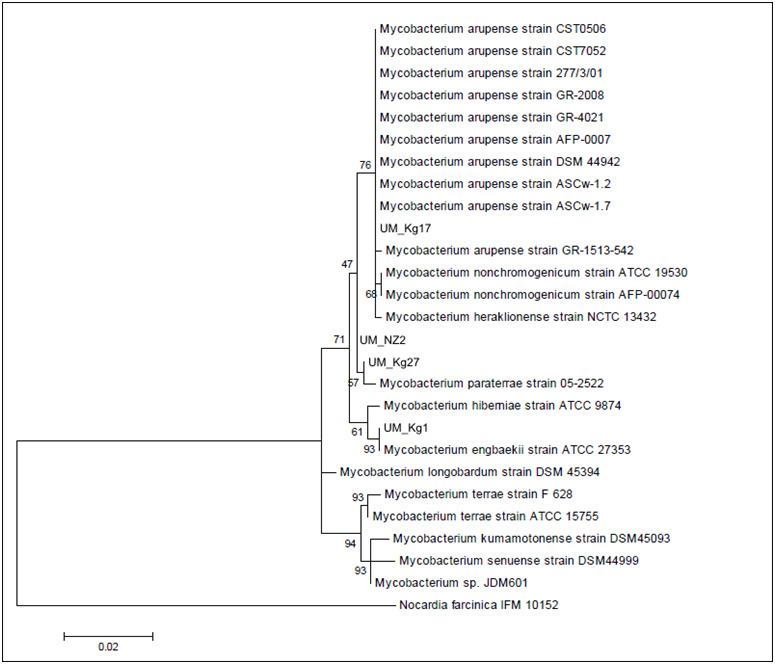

Supplement: S1 Fig — (TIF) [file pone.0120789.s001.tif]

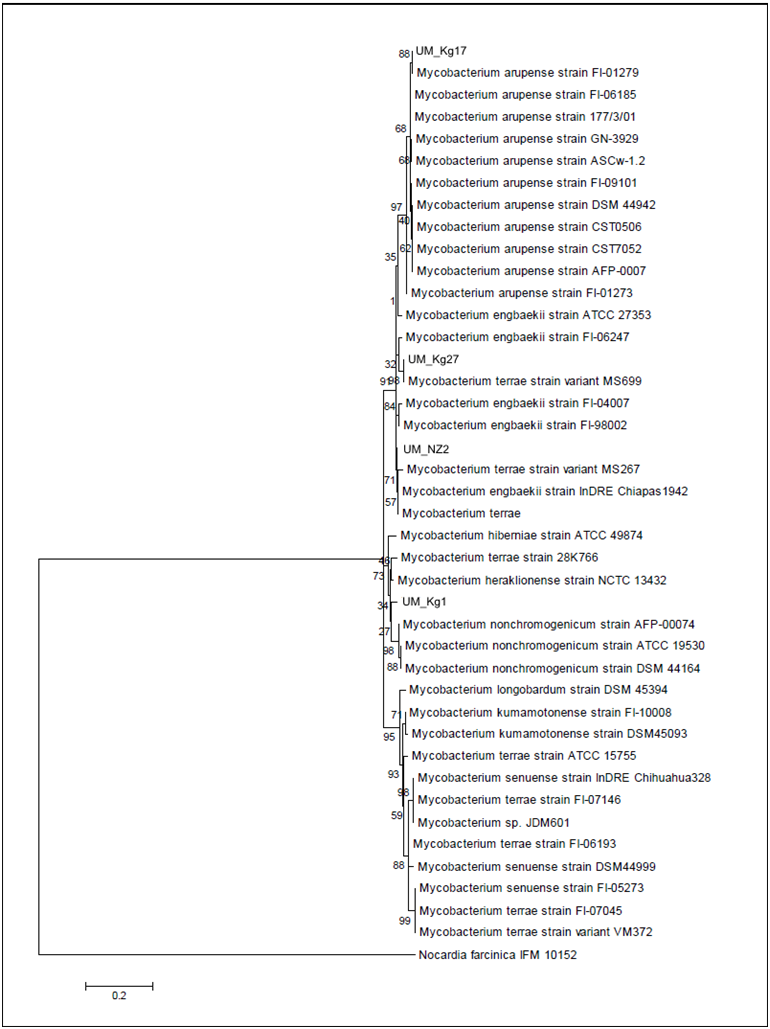

Supplement: S2 Fig — (TIF) [file pone.0120789.s002.tif]

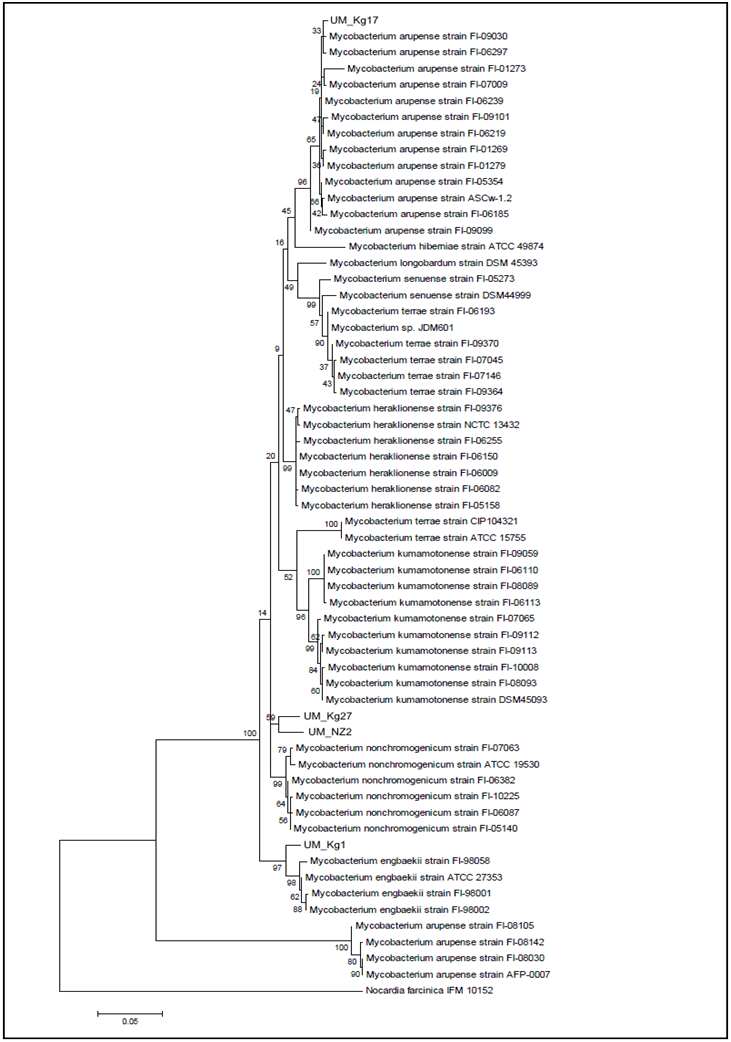

Supplement: S3 Fig — (TIF) [file pone.0120789.s003.tif]
