# Supplementary material for: Identification of New Genomospecies in the Mycobacterium terrae Complex
Source: PLoS One. 2015 Apr 1;10(4):e0120789. doi: 10.1371/journal.pone.0120789 (PMC4382200; doi:10.1371/journal.pone.0120789)
Supplement: S1 Table — (DOCX) [file pone.0120789.s004.docx]

S1 Table. Details of single gene amplifications by PCR

| **Gene** | **Primer sequence** | **Location in gene** | **Amplicon size** | **Reference** |
| --- | --- | --- | --- | --- |
| 16S rRNA | 27f (AGAGTTTGATCMTGGCTCAG)  907r (CCGTCAATTCMTTTRAGTTT) | nt8-nt926 | 918bp | Lane, 1991 (19) |
| *hsp65* | TB11 (ACCAACGATGGTGTGTCCAT)  TB12 (CTTGTCGAACCGCATACCCT) | nt145-nt585 | 440bp | Telenti et al., 1993 (21) |
| *rpoB* | MycoF (GGCAAGGTCACCCCGAAGGG)  MycoR (AGCGGCTGCTGGGTGATCATC) | nt2592-nt3316 | 724bp | Adekambi et al., 2003 (20) |
