# Supplementary material for: Identification of New Genomospecies in the Mycobacterium terrae Complex
Source: PLoS One. 2015 Apr 1;10(4):e0120789. doi: 10.1371/journal.pone.0120789 (PMC4382200; doi:10.1371/journal.pone.0120789)
Supplement: S2 Table — (DOCX) [file pone.0120789.s005.docx]

S2 Table. Accession numbers for genome sequences used in the phylogenetic analyses based on

SNPs and orthologous genes

| **Strains** | **Accession number** |
| --- | --- |
| *Mycobacterium smegmatis* str. MC2 155 | NC_008596 |
| *Mycobacterium fortuitum* subsp. *fortuitum* DSM 46621 | ALQB00000000 |
| *Mycobacterium septicum* DSM 44393 | CBMO000000000 |
| *Mycobacterium chelonae* 1818 | JAOI00000000 |
| *Mycobacterium abscessus* subsp. *bolletii* 50594 | NC_021282 |
| *Mycobacterium* sp. JDM601 | NC_015576 |
| *Mycobacterium avium* subsp. *paratuberculosis* K-10 | NC_002944 |
| *Mycobacterium avium* 104 | NC_008595 |
| *Mycobacterium intracellulare* ATCC 13950 | NC_016946 |
| *Mycobacterium parascrofulaceum* ATCC BAA-614 | ADNV00000000 |
| *Mycobacterium ulcerans* Agy99 | NC_008611 |
| *Mycobacterium marinum* M | NC_010612 |
| *Mycobacterium liflandii* 128FXT | NC_020133 |
