# Supplementary material for: Identification of New Genomospecies in the Mycobacterium terrae Complex
Source: PLoS One. 2015 Apr 1;10(4):e0120789. doi: 10.1371/journal.pone.0120789 (PMC4382200; doi:10.1371/journal.pone.0120789)
Supplement: S3 Table — (DOCX) [file pone.0120789.s006.docx]

S3 Table. Illumina Hiseq 2500 assembly details for four UM strains of mycobacteria

| **Strain** | **No. of Paired-end Reads** | **Genome Size** | **N50** | **No. of Contigs** | **No. of Protein CDS** | **No. of rRNA** | **No. of tRNA** | **%G+C** |
| --- | --- | --- | --- | --- | --- | --- | --- | --- |
|  |  |  |  |  |  |  |  |  |
| UM_Kg1 | 24,102,740 | 4,518,317 | 25,649 | 414 | 4216 | 3 | 51 | 68.5 |
| UM_Kg17 | 6,080,448 | 4,237,963 | 67,651 | 192 | 3923 | 3 | 61 | 67.6 |
| UM_Kg27 | 5,107,720 | 4,505,725 | 25,337 | 397 | 4233 | 5 | 54 | 67.5 |
| UM_NZ2 | 6,088,116 | 5,067,383 | 28,451 | 442 | 4811 | 3 | 54 | 67.4 |
