# Supplementary material for: Identification of New Genomospecies in the Mycobacterium terrae Complex
Source: PLoS One. 2015 Apr 1;10(4):e0120789. doi: 10.1371/journal.pone.0120789 (PMC4382200; doi:10.1371/journal.pone.0120789)
Supplement: S4 Table — (DOCX) [file pone.0120789.s007.docx]

S4 Table. Characteristics of prophages in UM strains

| **Strain** | **Intact** | **Incomplete** | **Total % prophage in genome (length)** | **Examples of key proteins** |
| --- | --- | --- | --- | --- |
| UM_Kg1 | 1 | - | 0.5 (24kb) | Type VII secretion system protein eccB1 |
| UM_Kg17 | 1 | 2 | 2.6 (112.3kb) | - General stress protein 39 - DNA-binding transcriptional repressor FabR - Putative copper export protein - mycofactocin system transcriptional regulator |
| UM_Kg27 | 1 | 2 | 1.3 (59.5kb) | - Group II intron-encoded protein ltrA - Phosphate import ATP-binding protein PstB |
| UM_NZ2 | 2 | 3 | 3.7 (187.6kb) | - Putative serine protease HtrA - Serine/threonine-protein kinase pknF - Flp pilus assembly protein CpaB |
