# Supplementary material for: Identification of New Genomospecies in the Mycobacterium terrae Complex
Source: PLoS One. 2015 Apr 1;10(4):e0120789. doi: 10.1371/journal.pone.0120789 (PMC4382200; doi:10.1371/journal.pone.0120789)
Supplement: S5 Table — (DOCX) [file pone.0120789.s008.docx]

S5 Table. 16S rRNA similarity matrix (%) between UM strains and reference strains

|  | UM_Kg1 | UM_Kg17 | UM_Kg27 | UM_NZ2 |
| --- | --- | --- | --- | --- |
| UM_Kg1 |  |  |  |  |
| UM_Kg17 | 98.36 |  |  |  |
| UM_Kg27 | 98.24 | 99.41 |  |  |
| UM_NZ2 | 98.36 | 99.53 | 99.88 |  |
| *Mycobacterium arupense* strain ASCw-1.7 | 98.36 | 100 | 99.41 | 99.53 |
| *Mycobacterium arupense* strain ASCw-1.2 | 98.36 | 100 | 99.41 | 99.53 |
| *Mycobacterium arupense* strain DSM 44942 | 98.36 | 100 | 99.41 | 99.53 |
| *Mycobacterium arupense* strain AFP-0007 | 98.36 | 100 | 99.41 | 99.53 |
| *Mycobacterium arupense* strain GR-4021 | 98.36 | 100 | 99.41 | 99.53 |
| *Mycobacterium arupense* strain GR-1513-542 | 98.24 | 99.88 | 99.3 | 99.41 |
| *Mycobacterium arupense* strain GR-2008 | 98.36 | 100 | 99.41 | 99.53 |
| *Mycobacterium arupense* strain 277/3/01 | 98.36 | 100 | 99.41 | 99.53 |
| *Mycobacterium arupense* strain CST0506 | 98.36 | 100 | 99.41 | 99.53 |
| *Mycobacterium arupense* strain CST7052 | 98.36 | 100 | 99.41 | 99.53 |
| *Mycobacterium engbaekii* strain ATCC 27353 | 100 | 98.36 | 98.24 | 98.36 |
| *Mycobacterium hiberniae* strain ATCC 9874 | 99.53 | 98.35 | 98.24 | 98.35 |
| *Mycobacterium kumamotonense* strain DSM45093 | 97.3 | 97.07 | 97.3 | 97.18 |
| *Mycobacterium nonchromoge*nicum strain ATCC 19530 | 98.12 | 98.94 | 98.35 | 98.47 |
| *Mycobacterium nonchromogenicum* strain AFP-00074 | 98.59 | 99.41 | 98.83 | 98.94 |
| *Mycobacterium paraterrae* strain 05-2522 | 98 | 99.18 | 99.77 | 99.65 |
| *Mycobacterium senuense* strain DSM44999 | 96.95 | 97.54 | 97.77 | 97.65 |
| *Mycobacterium terrae* strain F 628 | 98.11 | 96.81 | 97.04 | 96.93 |
| *Mycobacterium terrae* strain ATCC 15755 | 98.71 | 97.42 | 97.65 | 97.54 |
| *Mycobacterium sp*. JDM601 | 98.36 | 97.54 | 97.77 | 97.65 |
| *Mycobacterium heraklionense* strain NCTC 13432 | 98.36 | 99.77 | 99.41 | 99.3 |
| *Mycobacterium longobardum* strain DSM 45394 | 97.89 | 98.24 | 98.59 | 98.47 |
| *Nocardia farcinica* IFM 10152 | 91.85 | 91.85 | 91.97 | 91.85 |
