# Supplementary material for: Identification of New Genomospecies in the Mycobacterium terrae Complex
Source: PLoS One. 2015 Apr 1;10(4):e0120789. doi: 10.1371/journal.pone.0120789 (PMC4382200; doi:10.1371/journal.pone.0120789)
Supplement: S6 Table — (DOCX) [file pone.0120789.s009.docx]

S6 Table. *hsp65* similarity matrix (%) between UM strains and reference strains

|  | UM_Kg1 | UM_Kg17 | UM_Kg27 | UM_NZ2 |
| --- | --- | --- | --- | --- |
| UM_Kg1 |  |  |  |  |
| UM_Kg17 | 92.98 |  |  |  |
| UM_Kg27 | 94.99 | 95.74 |  |  |
| UM_NZ2 | 94.99 | 95.99 | 97.74 |  |
| *Mycobacterium arupense* strain FI-01273 | 93.73 | 98.75 | 96.49 | 97.24 |
| *Mycobacterium arupense* strain FI-09101 | 93.73 | 99.25 | 96.49 | 96.74 |
| *Mycobacterium arupense* strain FI-06185 | 93.48 | 99.5 | 96.24 | 96.49 |
| *Mycobacterium arupense* strain FI-01279 | 92.98 | 100 | 95.74 | 95.99 |
| *Mycobacterium arupense* strain GN-3929 | 93.73 | 99.25 | 95.99 | 96.24 |
| *Mycobacterium arupense* strain DSM 44942 | 93.98 | 99 | 96.74 | 96.99 |
| *Mycobacterium arupense* strain 177/3/01 | 93.48 | 99.5 | 96.24 | 96.49 |
| *Mycobacterium arupense* strain CST0506 | 93.98 | 99 | 96.74 | 96.99 |
| *Mycobacterium arupense* strain CST7052 | 93.98 | 99 | 96.74 | 96.99 |
| *Mycobacterium arupense* strain ASCw-1.2 | 93.44 | 99.21 | 95.8 | 96.06 |
| *Mycobacterium arupense* strain AFP-0007 | 93.62 | 98.94 | 96.54 | 96.81 |
| *Mycobacterium engbaekii* strain InDRE Chiapas1942 | 94.74 | 95.74 | 97.49 | 99.75 |
| *Mycobacterium engbaekii* strain FI-06247 | 94.74 | 95.24 | 97.74 | 98.25 |
| *Mycobacterium engbaekii* strain ATCC 27353 | 94.74 | 95.74 | 96.99 | 98.25 |
| *Mycobacterium engbaekii* strain FI-04007 | 96.49 | 94.74 | 96.99 | 98.25 |
| *Mycobacterium engbaekii* strain FI-98002 | 95.99 | 95.24 | 97.49 | 98.75 |
| *Mycobacterium hiberniae* strain ATCC 49874 | 97.49 | 93.73 | 95.49 | 94.74 |
| *Mycobacterium kumamotonense* strain FI-10008 | 94.74 | 91.73 | 93.73 | 93.23 |
| *Mycobacterium kumamotonense* strain DSM45093 | 94.49 | 91.48 | 93.48 | 92.98 |
| *Mycobacterium nonchromogenicum* strain AFP-00074 | 97.49 | 93.23 | 94.49 | 94.74 |
| *Mycobacterium nonchromogenicum* strain ATCC 19530 | 96.99 | 92.73 | 94.49 | 94.24 |
| *Mycobacterium nonchromogenicum* strain DSM 44164 | 96.99 | 92.73 | 94.49 | 94.24 |
| *Mycobacterium senuense* strain InDRE Chihuahua328 | 92.73 | 89.72 | 91.73 | 91.23 |
| *Mycobacterium senuense* strain FI-05273 | 91.73 | 88.97 | 90.73 | 90.98 |
| *Mycobacterium senuense* strain DSM44999 | 93.48 | 90.73 | 92.48 | 92.48 |
| *Mycobacterium terrae* strain FI-07146 | 92.73 | 89.72 | 91.73 | 91.23 |
| *Mycobacterium terrae* strain FI-06193 | 93.98 | 90.48 | 92.98 | 92.48 |
| *Mycobacterium terrae* strain FI-07045 | 92.23 | 89.47 | 91.23 | 91.48 |
| *Mycobacterium terrae* | 94.74 | 95.74 | 97.49 | 99.75 |
| *Mycobacterium terrae* strain 28K766 | 97.24 | 92.98 | 95.49 | 95.49 |
| *Mycobacterium terrae* strain ATCC 15755 | 94.24 | 91.23 | 93.23 | 93.23 |
| *Mycobacterium terrae* strain variant MS267 | 94.24 | 94.74 | 96.49 | 98.75 |
| *Mycobacterium terrae* strain variant MS699 | 94.99 | 95.74 | 100 | 97.74 |
| *Mycobacterium terrae* strain variant VM372 | 91.73 | 88.97 | 90.73 | 90.98 |
| *Mycobacterium sp.* JDM601 | 92.73 | 89.72 | 91.73 | 91.23 |
| *Mycobacterium heraklionense* strain NCTC 13432 | 97.74 | 93.73 | 95.74 | 95.74 |
| *Mycobacterium longobardum* strain DSM 45394 | 94.99 | 91.98 | 94.49 | 93.98 |
| *Nocardia farcinica* IFM 10152 | 68.67 | 67.17 | 67.67 | 67.42 |
