# Supplementary material for: Identification of New Genomospecies in the Mycobacterium terrae Complex
Source: PLoS One. 2015 Apr 1;10(4):e0120789. doi: 10.1371/journal.pone.0120789 (PMC4382200; doi:10.1371/journal.pone.0120789)
Supplement: S7 Table — (DOCX) [file pone.0120789.s010.docx]

S7 Table. *rpoB* similarity matrix (%) between UM strains and reference strains

|  | UM_Kg1 | UM_Kg17 | UM_Kg27 | UM_NZ2 |
| --- | --- | --- | --- | --- |
| UM_Kg1 | 100 | 94.21 | 95.99 | 94.81 |
| UM_Kg17 | 94.21 | 100 | 94.81 | 94.96 |
| UM_Kg27 | 95.99 | 94.81 | 100 | 97.18 |
| UM_NZ2 | 94.81 | 94.96 | 97.18 | 100 |
| *Mycobacterium arupense* strain AFP-0007 | 87.67 | 86.32 | 88.27 | 87.67 |
| *Mycobacterium arupense* strain ASCw-1.2 | 94.51 | 99.26 | 95.1 | 94.96 |
| *Mycobacterium arupense* strain FI-01269 | 94.66 | 99.26 | 95.4 | 95.25 |
| *Mycobacterium arupense* strain FI-01273 | 94.36 | 97.77 | 94.51 | 94.96 |
| *Mycobacterium arupense* strain FI-01279 | 94.51 | 99.41 | 95.25 | 95.1 |
| *Mycobacterium arupense* strain FI-05354 | 94.36 | 99.41 | 94.96 | 94.81 |
| *Mycobacterium arupense* strain FI-06185 | 94.21 | 99.11 | 94.81 | 94.66 |
| *Mycobacterium arupense* strain FI-06219 | 94.81 | 99.41 | 95.1 | 94.96 |
| *Mycobacterium arupense* strain FI-06239 | 94.66 | 99.55 | 95.25 | 95.1 |
| *Mycobacterium arupense* strain FI-06297 | 94.36 | 99.55 | 94.96 | 94.81 |
| *Mycobacterium arupense* strain FI-07009 | 94.36 | 99.26 | 94.96 | 94.81 |
| *Mycobacterium arupense* strain FI-08030 | 87.67 | 86.32 | 88.27 | 87.67 |
| *Mycobacterium arupense* strain FI-08105 | 87.37 | 86.32 | 88.27 | 87.37 |
| *Mycobacterium arupense* strain FI-08142 | 87.37 | 86.32 | 87.97 | 87.37 |
| *Mycobacterium arupense* strain FI-09030 | 94.36 | 99.55 | 94.96 | 94.81 |
| *Mycobacterium arupense* strain FI-09099 | 95.1 | 98.81 | 95.7 | 95.55 |
| *Mycobacterium arupense* strain FI-09101 | 94.51 | 99.11 | 94.96 | 94.81 |
| *Mycobacterium engbaekii* strain ATCC 27353 | 98.07 | 94.36 | 95.4 | 94.96 |
| *Mycobacterium engbaekii* strain FI-98001 | 97.63 | 94.21 | 95.25 | 94.81 |
| *Mycobacterium engbaekii* strain FI-98002 | 97.77 | 94.36 | 95.4 | 94.96 |
| *Mycobacterium engbaekii* strain FI-98058 | 97.63 | 94.51 | 94.96 | 94.81 |
| *Mycobacterium heraklionense* strain FI-05158 | 95.85 | 95.7 | 97.03 | 96.44 |
| *Mycobacterium heraklionense* strain FI-06009 | 95.85 | 95.99 | 97.33 | 96.74 |
| *Mycobacterium heraklionense* strain FI-06082 | 95.7 | 95.85 | 97.18 | 96.59 |
| *Mycobacterium heraklionense* strain FI-06150 | 95.85 | 95.99 | 97.33 | 96.74 |
| *Mycobacterium heraklionense* strain FI-06255 | 95.85 | 95.85 | 97.03 | 96.59 |
| *Mycobacterium heraklionense* strain FI-09376 | 96.14 | 95.85 | 97.03 | 96.44 |
| *Mycobacterium heraklionense* strain NCTC 13432 | 95.99 | 95.55 | 96.88 | 96.29 |
| *Mycobacterium hiberniae* strain ATCC 49874 | 96.14 | 94.96 | 94.36 | 94.07 |
| *Mycobacterium kumamotonense* strain DSM45093 | 95.55 | 94.51 | 95.25 | 95.55 |
| *Mycobacterium kumamotonense* strain FI-06110 | 94.66 | 93.92 | 94.66 | 94.07 |
| *Mycobacterium kumamotonense* strain FI-06113 | 94.51 | 93.92 | 94.66 | 94.07 |
| *Mycobacterium kumamotonense* strain FI-07065 | 95.99 | 94.81 | 95.4 | 95.7 |
| *Mycobacterium kumamotonense* strain FI-08089 | 94.66 | 93.92 | 94.66 | 94.07 |
| *Mycobacterium kumamotonense* strain FI-08093 | 95.55 | 94.51 | 95.25 | 95.55 |
| *Mycobacterium kumamotonense* strain FI-09059 | 94.66 | 93.92 | 94.66 | 94.07 |
| *Mycobacterium kumamotonense* strain FI-09112 | 95.4 | 94.36 | 95.4 | 95.7 |
| *Mycobacterium kumamotonense* strain FI-09113 | 95.55 | 94.51 | 95.25 | 95.55 |
| *Mycobacterium kumamotonense* strain FI-10008 | 95.4 | 94.36 | 95.4 | 95.7 |
| *Mycobacterium longobardum* strain DSM 45393 | 94.51 | 95.55 | 95.1 | 95.25 |
| *Mycobacterium nonchromogenicum* strain ATCC 19530 | 96.29 | 94.81 | 97.03 | 97.03 |
| *Mycobacterium nonchromogenicum* strain FI-05140 | 96.44 | 95.4 | 97.03 | 97.03 |
| *Mycobacterium nonchromogenicum* strain FI-06087 | 96.29 | 95.55 | 96.88 | 97.18 |
| *Mycobacterium nonchromogenicum* strain FI-06382 | 96.59 | 95.25 | 97.18 | 97.18 |
| *Mycobacterium nonchromogenicum* strain FI-07063 | 96.74 | 94.96 | 97.18 | 96.88 |
| *Mycobacterium nonchromogenicum* strain FI-10225 | 96.29 | 95.25 | 97.18 | 97.18 |
| *Mycobacterium senuense* strain DSM44999 | 94.21 | 95.25 | 94.66 | 94.81 |
| *Mycobacterium senuense* strain FI-05273 | 94.81 | 95.25 | 95.1 | 94.96 |
| *Mycobacterium sp*. JDM601 | 94.36 | 95.4 | 94.66 | 95.1 |
| *Mycobacterium terrae* strain ATCC 15755 | 93.03 | 94.07 | 94.66 | 94.07 |
| *Mycobacterium terrae* strain CIP104321 | 93.03 | 94.07 | 94.66 | 94.07 |
| *Mycobacterium terrae* strain FI-06193 | 94.36 | 95.4 | 94.66 | 95.1 |
| *Mycobacterium terrae* strain FI-07045 | 94.21 | 95.7 | 94.66 | 95.1 |
| *Mycobacterium terrae* strain FI-07146 | 94.07 | 95.55 | 94.51 | 94.96 |
| *Mycobacterium terrae* strain FI-09364 | 94.21 | 95.4 | 94.66 | 95.1 |
| *Mycobacterium terrae* strain FI-09370 | 94.07 | 95.7 | 94.66 | 95.1 |
| *Nocardia farcinica* IFM 10152 | 84.06 | 83.91 | 83.91 | 84.06 |
